# Praise Your Wife

**Published:** 1881-01

**Authors:** 


### PRAISE YOUR WIFE.

Praise your wife, man ; for pity's sake, praise your wife when she deserves it ! It won't injure her any, though it may frighten her some from its strangeness. If you wish to make and keep her happy, give her a loving word occasionally. If she takes pains to make you something pretty, don't take it with only :

"Yes, it is very pretty. Won't you hand me my paper ?"

It will cost you only a moment's time to kiss her and tell her she is the best wife in town. You will find it to be a paying investment—one which will yield you a large return in increased care and willing labor for your comfort. Loving praise will lighten labor wonderfully, and it should be freely bestowed. A case in point :

I called on a friend one day and found her up to her eyes in work. "O, dear," she said, "this is one of my days ; everything goes wrong, and I haven't got a thing done ?"

"Let me help you," I said.

"No, no," she replied, gently pushing me into the sitting-room, "I'm going to leave everything and rest a while ; but I must just wipe up this slop first," pointing to an ugly spot which disfigured the pretty oil-cloth.

Just as she stopped to do it her husband came in ; he didn't see me, but went straight to his wife. One quick lift, and he placed her on her feet, and taking the cloth from her hand, wiped up the spot himself.

"There, busy-bee," he said, "you've done enough to-day. You tired yourself all out getting my favorite dinner, Now I think I'd leave the rest till to morrow."

I spoke to him then, and he sat with me a few minutes before going down town. Shortly after my friend came in, looking very much amused.

"I guess I was in the dumps," she said, laughing, "for I've finished ; and everything has gone swimmingly since E. came in."
—*Anna Edwards.*

"I HAVE three children who are the very image of myself." "I pity the youngest," replied his interlocutor. "Why ?" "Because he is the one who will have to resemble you the longest."

### HIGH HEELS.

NATURE has doubtless formed the feet so that locomotion is most natural and least fatiguing when the ball of the foot and the heel touch the ground in the same horizontal plane. Any deviation from this position, as by unduly elevating the heels, causes a strain on the muscles, which will sooner or later impair their elasticity and cause the gait to be shuffling and awkward. The forcible impingement and wedging of the feet into the toes of the shoes at every step is the real cause of corns and bunions. In its natural position, the weight of the body is supported upon the elastic arch between the heel and the ball of the foot, and the foot rests upon these two points. The elasticity of this arch is essential to an easy and graceful carriage. If high heels are worn in walking, the heel reaches the ground first, and the strain is unequally distributed ; when, however, in addition to being too high, the heel of the shoe is placed nearly under the center of the foot, the evil is greatly aggravated, because the weight of the body then rests upon the blood vessels and nerves of the instep, which are totally unfit to support it. The immediate evils resulting from this pressure are great fatigue, nausea, vertigo and palpitation of the heart, which may result in permanent invalidity.

### MORE CLIFF DWELLERS.

Colonel Stephenson, of the United States geological survey, has turned another page in the long-sealed volume of American antiquities. A large village of cliff dwellers has been discovered between the Jemez Mountains and the Rio Grande River, in New Mexico. The cliffs rise to a height of from fifty to five hundred feet. Some of them contain two, some three and others as many as five lines of dwellings, rising line above line, and, back toward the mountain, tier above tier. The houses on the top of the cliff in the abandoned city are rectangular in form, but the caves are circular, being ten or fifteen feet in diameter, with arched roofs. Within the excavations are numerous small rooms. Before each line of dwelling, there appears to have been pavements sometimes four or five feet in width, on the broadest of which Colonel Stephenson found imprints of feet. Many pictures and hieroglyphics adorn the face of the rock.

Is life worth living ! That depends on the *liver*.

